# Spider Neurotoxins, Short Linear Cationic Peptides and Venom Protein Classification Improved by an Automated Competition between Exhaustive Profile HMM Classifiers

**DOI:** 10.3390/toxins9080245

**Published:** 2017-08-08

**Authors:** Dominique Koua, Lucia Kuhn-Nentwig

**Affiliations:** 1 Departement Agriculture et Ressources Animals, Institut National Polytechnique Félix Houphouet-Boigny, BP 1093 Yamoussoukro, Ivory Coast; 2 Institute of Ecology and Evolution, University of Bern, Baltzerstrasse 6, 3012 Bern, Switzerland; lucia.kuhn@iee.unibe.ch

**Keywords:** spider, toxin, classification, profile HMM, hmmcompete, machine learning

## Abstract

Spider venoms are rich cocktails of bioactive peptides, proteins, and enzymes that are being intensively investigated over the years. In order to provide a better comprehension of that richness, we propose a three-level family classification system for spider venom components. This classification is supported by an exhaustive set of 219 new profile hidden Markov models (HMMs) able to attribute a given peptide to its precise peptide type, family, and group. The proposed classification has the advantages of being totally independent from variable spider taxonomic names and can easily evolve. In addition to the new classifiers, we introduce and demonstrate the efficiency of *hmmcompete*, a new standalone tool that monitors HMM-based family classification and, after post-processing the result, reports the best classifier when multiple models produce significant scores towards given peptide queries. The combined used of *hmmcompete* and the new spider venom component-specific classifiers demonstrated 96% sensitivity to properly classify all known spider toxins from the UniProtKB database. These tools are timely regarding the important classification needs caused by the increasing number of peptides and proteins generated by transcriptomic projects.

## 1. Introduction

Spiders have evolved a very broad range of venom peptides and proteins designed for predatory and defensive purposes [1,2]. Despite the intensive investigation of spider toxins [3,4,5,6] and the fast-growing number of new spider venom peptides and proteins due to transcriptomic studies, neither a uniform nor a widely-accepted family classification system has emerged.

Currently, the ToxProt database, the animal toxin annotation project of UniProt [7], represent with Arachnoserver [8] the main repositories of spider venom-derived sequences. ToxProt contains 1379 manually-reviewed entries (status as of 20 April 2017). In contrast to ArachnoServer, which completely relies on a rational naming system [9] and, therefore, sort sequences according to spider taxonomic families, UniProtKB includes a peptide family classification annotation based on InterPro signatures and, more specifically, to Pfam profile HMMs [10,11]. This taxonomy-independent family classification complements the naming system and appears useful to characterize new peptides [12]. However, a closer look at this UniProtKB spider peptide family classification indicates two major problems.

First, various types of peptide family name types are found and different approaches could be assumed to explain the origin of adopted peptide family names. A first category of family names concerns cryptic or generic terms (e.g., Shiva superfamily, Magi-1 superfamily, spider insecticidal toxin) which do not provide any clue about the sequence origin or function. Indeed, naming a spider toxin family “insecticidal family” is helpless and may suggest that toxins not belonging to that family do not have insecticidal activity. A second category is represented by names that refer to the taxonomic family of the spider. Most of the time, names of the taxonomic family whose peptides were first described are used (e.g., Huwentoxin-1 superfamily, Plectoxin superfamily, CsTx family, etc.). These names have more a historical origin than a scientific justification. For example, the name “Huwentoxin” was originally given to sequences from the Chinese bird spider *Ornithoctonus huwenum*, whose name has meanwhile changed to *Cyriopagopus schmidti*. In addition, “huwentoxin” intuitively makes one think of Theraphosidae species. However, this “Huwentoxin family” currently includes peptides from Barychelidae, Ctenidae, Sparassidae, and Theraphosidae. A third category of family names refers to peptide activity combined with spider taxonomic family names, or even to peptide structures (for example, beta/delta-agatoxin family, venom kunitz-type family). This type of family name gives a first hint about the scientific background that justified sequence grouping in such families. However, some families have been named using a “U” prefix (e.g., U6- to U10-lycotoxin families). This “U” stands for “unknown function”. Therefore, a family of peptides having an unknown function may contain various peptides having different, even if unknown, functions. Finally, some family names seem related to the classifier used for sequence annotation. For example, the spider agouti family is named according to the agouti domain signature (IPR007733) from InterPro. This signature name has initially been assigned based on a mammal name. This kind of name transfer from mammalians to spiders increases the confusion.

The second problem with UniProt spider toxin classification in its current state is the hierarchical heterogeneity. Indeed, in some cases, a superfamily/family organization is chosen and, in some others, a family/subfamily subdivision is used. In any case, no clear definition can be found to explain this hierarchy.

As indicated above, the ToxProt family classification is based on InterPro signatures. Currently, 35 different InterPro signatures (among which 23 are Pfam HMMs) are used alone or in combination to classify 1036 spider toxins (Table 1). Another 229 peptides from the database are classified, but not related to any InterPro signature (Table 2). This classification was probably based on sequence similarity. The remaining 114 reviewed sequences are not associated to any peptide family or InterPro signature. Furthermore, despite their great importance, spider venom proteins are nearly excluded from the current classification system. Only a few are reported: arthropod phospholipases D (only from *Loxosceles arizonica)* and proteases (five entries found). A deeper analysis of the current InterPro-based classification indicated two limitations for currently-used signatures. First, sequences matching a single InterPro signature are spread among various peptide families (Table 1). This indicates that either family boundaries are not clearly defined or that InterPro signatures are not sensitive enough. In any case, this situation obviously makes it difficult to classify a new peptide and requires improvement. Second, many known peptides could not be assigned to a valuable class based on existing signatures: currently 343 out of 1379 sequences (~25%) are not related to any signature (Table 2). This situation suggests that more classifiers are needed and/or the sensitivity of current classifiers has to be improved.

The table shows that sequences matching a single InterPro signature are spread among various peptide families. This indicates that either family boundaries are not clearly defined or that InterPro signature are not sensitive enough.

From these observations, it appears that, on one hand, the majority of currently-used family names are confusing for non-expert users. A better family organization system should be adopted to avoid confusion and provide helpful hints for further analyses of these toxins. On the other hand, the current classifier set (InterPro signatures) requires some refinement to increase their sensitivity. In addition to these limitations, observed discrepancies between InterPro signatures and the manually-assigned family also require analysis to avoid ambiguity.

As alternative to InterPro or Pfam, Generalist web tools like ToxClassifier (http://bioserv7.bioinfo.pbf.hr/ToxClassifier/) or ClanTox (http://www.clantox.cs.huji.ac.il/) [13] exist. They are based on machine learning (neural network and HMMs). They are intended to predict if a given peptide is an animal toxin sequence or not. Even if this is a good first step after sequence acquisition, their practical usage to obtain a precise family classification of sequences is not possible. Therefore, neither ToxClassifier nor ClanTox can be regarded as valuable classification tools for spider venom components.

From a computational point of view, an ideal classification system should mainly consider the structure of peptide. Concerning spider venom peptides, a commonly-used classification system is based on the number and distribution of cysteine residues, as well as the disulfide framework [14]. Such classification systems focus mainly on the structural organization of peptides since the disulfide framework directs the 3D fold of peptides and is, therefore, highly correlated with the function. The main difficulty in applying this system to spider toxin sequences is the highly variable number of residues found between pairs of cysteine. For example, a majority of spider neurotoxic peptides share the Inhibitor Cystine Knot (ICK) motif, which is composed of six cysteine residues with three disulfide bridges (C1–C4, C2–C5, and C3–C6). The inter-cysteine distribution of these ICK peptides is given by the pattern X(6)-C-X(4,6)-C-X(4,9)-C-C-X(2,10)-C-X(3,14)-C-X(1,16), where X represents any amino acid followed by the number of potential residue one can observe [14]. This variability makes it difficult to design a unique valuable classifier. Indeed, a classifier trying to embrace all ICK peptides would be poorly sensitive because of this high variability. At most, like that adopted for Pfam HMMs, one should divide the ICK-containing peptides in small, structurally-conserved groups.

In this study, we propose new spider-specific profile HMMs for family classification of spider venom-derived peptides and proteins. These classifiers are based on sequence primary structure. These new classifiers allow properly and reliably distributing 96% of known spider toxin sequences in their correct classification level. In addition to classifiers, we introduce the *hmmcompete* tool. This standalone program helps to monitor the HMM-based classification, to internally post-process the initial result and, finally, select from a profile HMM database the model giving the best classification for a given amino acid sequence. In this next generation genomic era, the sequencing approach will be more intensively used in biological studies for exhaustive screening of animal venom gland transcriptomes. Numerous spider toxin sequences are being generated by this method and already require automatic classifiers able to quickly and reliably assign a structural classification. This will help to predict and/or identify new, interesting candidates for further studies, like pharmacological development or molecular phylogeny. The proposed classifiers are, therefore, timely and could serve as valuable tools to provide an initial hint toward further investigations of incoming spider venom-derived peptides.

## 2. Results

### 2.1. New Classification System and Exhaustive Classifiers for Spider Venom Components

For profile HMM construction, all known spider venom-derived sequences were extracted from ToxProt (UniProtKB release 2017_04, on 20 April 2017). Sequences were divided into structural conservation groups based (i) on the distribution of cysteine residues; (ii) on the number of amino acid residues between conserved cysteine; and (iii) on amino acid properties (charge, hydrophobicity, size). Sequences in structural conservation groups were aligned to generate multiple sequence alignment (MSA) files that were manually cleaned. Cleaned MSAs were then used to build new classifiers for spider venom peptides. These new classifiers were validated by using ToxProt reviewed sequences as a testing set. Our classifier predictions were compared to ToxProt manual annotation and available InterPro and Pfam signatures. We finally selected 219 new profile HMMs (Table 3, Supplementary File F1: ekenda_class.hmm) that allow for reliable classification of spider venom components using a three-level classification schema: 21 models for short linear cationic peptides, 170 models for neurotoxins, and 28 models for venom proteins.

**Figure 1:**
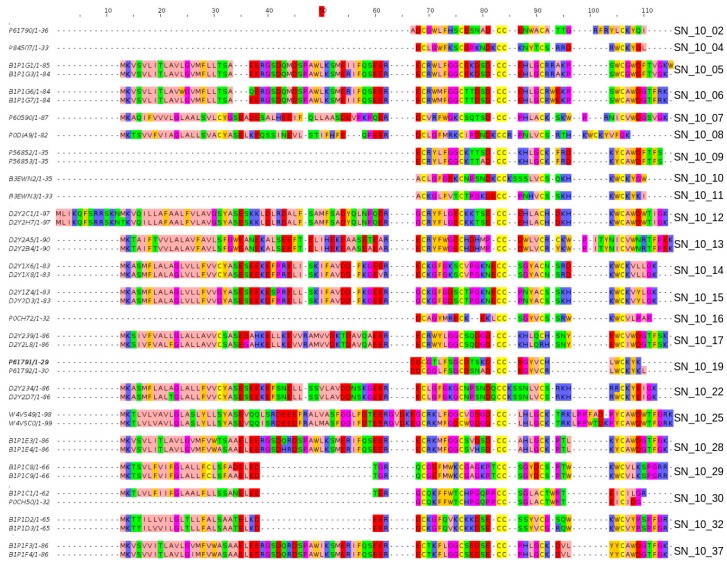
The new classifiers are able to produce very sharp discrimination between closely-related structural compositions. While all shown sequences share the cysteine framework C-C-CC-C-C, match the Pfam model Toxin_12 (PF07740), and are annotated in ToxProt as members of the huwentoxin-1 family, our new classifiers were able to separate them between various Spider Neurotoxin family 10 groups, each group representing a specific structural variation.

We decided to follow a neutral family naming system for the newly-built classifiers and their related classification levels. Our first classification level concerns the general toxin sequence type. In the current case, since the term “Toxin” is too broad and common, so we propose using a two-letter abbreviation for the sequence type that will represent the first part of the new class names. We used SN for spider neurotoxins (these peptides have cysteine residues), SC for short cationic peptides (concerns short linear peptides that often exhibit cytolytic activity), and VP for venom proteins including venom enzymes, peptidase inhibitors, and proteins with unknown function. The sequence type will be followed by an integer value (represented by two digits) representing the second classification level. This level 2 order could be considered as the peptide family. A numerical value (represented by two digits) will represent the third classification level, as well as the third part of the name. This third level represents the structurally consistent division of the peptide family (Figure 1). The three parts (levels) of classifier names are separated by an underscore character (_). For example SN_10_00 represents the spider neurotoxin family 10 (previously named Huwentoxin-1 family); SN_10_01, SN_10_02, and SN_10_03, respectively, represent the first, second, and third group inside the spider neurotoxin family 10.

Our level 2 and level 3 numbers are strictly ordinal values and do not represent any kind of biological relationship inside the concerned classification level. Only level 3 sharing the same level 2 value could be considered structurally related since they share sequence similarities. For level 2 classification, we mostly relied on the currently-used InterPro (and/or Pfam) signatures when they were available. These signatures can, more or less, explain the global classification system used in ToxProt even if resulting family names are confusing. For level 3, the zero order (00) was used to characterize a global level 2 classifier (a classifier that allows to fish out (almost) all sequences distributed among associated level 3 groups). When no subdivision was necessary to properly classify sequences in a given level 2, only the level 3 model with index 00 (xx_nn_00) was proposed instead of index 01 (xx_nn_01). Using xx_nn_01 would indicate that we consider all available sequences as the first subgroup, but we do not. Using the 00-order (xx_nn_00) properly indicates that the considered profile HMM isolate all known sequences of the considered level 2 family. When more sequences are available, it will be possible to split the considered level 2 into several groups and create more specific profiles that will be assigned xx_nn_01, xx_nn_02, xx_nn_03, and successive level 3 orders. The initial xx_nn_00 model will hopefully still continue to isolate all sequences belonging to these new groups and, therefore, effectively play its role of a global classifier.

When a more precise classifier is proposed, a match with the level 3 model with index 00 (xx_nn_00) indicates the possibility to design a new level 3 classifier in the corresponding family. This assumption guided the subdivision of families into consistent structural groups.

Since our new classification system is merely ordinal, to facilitate the transition step from the old to the new system, previous ToxProt annotation are indicated in the table and added as description of the new profile HMMs (DESC field). A detailed list with the ToxProt family corresponding to each new profile HMM is provided as supplementary material (File F2: classif_level.xls).

### 2.2. Spider Peptide Classification is Improved

Moving from 22 (Pfam classifiers) to 219 new profile HMMs allowed achieving a complete and more precise classification of spider venom-derived sequence (including toxins, peptides, and proteins). To the best of our knowledge, this study specifically provides the first exhaustive set of machine learning classifiers for spider small linear cationic peptides mostly exhibiting cytolytic activity, spider neurotoxins, and venom proteins (including enzymes). For instance, concerning spider neurotoxic peptides, we propose the first complete profile HMM-based classification with 40 family classifiers instead of the current 19 Pfam HMMs and 170 level 3 classifiers. Compared to existing Pfam classifiers, our new models are mainly intended to achieve a more precise classification of spider toxin sequences. Concerning venom proteins, we provide 28 new valuable classifiers for sequences families known to be widespread in spider venom glands, and also, identified in various venomous arthropods. The structures of these proteins with partial unknown function are of great interest since they were shown to act as venom peptide precursor processing enzymes (signal peptidase, venom serine protease, protein disulfide isomerase, and peptidylglycine alpha-amidating monooxygenase), protease inhibitors, or toxic-acting enzymes. These proteins now have their respective HMM descriptors to facilitate their identification and annotation.

In order to evaluate the sensitivity of the proposed profile HMMs, we performed three separate classification tests on ToxProt sequences (using our new *hmmcompete* tool described below): (i) the existing Pfam HMMs were concatenated in a single .hmm file; (ii) the new spider toxins HMMs proposed in this study were concatenated in a single file and, finally; (iii) we combined Pfam HMMs and our new profile HMMs. Each profile dataset was used to perform the classification of ToxProt spider sequences. In each case, for every sequence, only the best classifier was reported (Supplementary File F8: ToxProt_classif_comparison.csv). When used in the context of sequence annotation by the Pfam internal algorithm, a manual cutoff of 20 is used. However, in this study, this cutoff was not considered, but only the best domain score was considered. This increased the number of sequences matched by these Pfam HMMs and explained the difference of sequence distribution compared to the ToxProt annotations (Table 3).

On one hand, considering the reviewed sequences from ToxProt, our new profile HMMs allowed the classification of 1334 sequences out of 1379 (97%) compared to Pfam HMMs (921/1379, 67%). When merged, all classifiers allowed assigning a classification to 1337 sequences, with three sequences only assigned thanks to Pfam Toxin_35 HMM. The combined classification test indicated that our models performed better (giving the best domain bit score) on 1266 classified sequences (94.7%) compared to Pfam HMM (71/1337 = 5.3%). For example, for the sequence A9XDG0, the new classifier SN_29_00 generated a score of 106.5 when the corresponding Pfam Toxin_35 only yielded 26.0. For the 71 sequences where a Pfam classifier was the better predictor, our models provide at least the same family classification: the family assigned by our level 2 models was equivalent to the ToxProt level 1 annotation (TPL1), or even better (more specific thanks to level 3 models). Furthermore, for the sequence P81885, the Pfam atracotoxin model yields a score of 83.1 when our new profile SN_06_00 produced a comparable score of 82.7.

When we also included non-reviewed entries from ToxProt, on a total of 1616 entries, our new models were able to assign a proper classification to 1563 (96.72%). As expected, the new classifiers are also performing well for sequences not used for the training step. Their predictive capacity makes them handy for the classification of new upcoming sequences from transcriptomic and/or proteomic projects.

On the other hand, Pfam HMMs currently associated to spider toxins could be considered as family classifiers (level 2) and the new profiles represent efficient tools to realize a level 3 distribution of sequences matched by these Pfam HMMs. However, it clearly appeared that, in many cases, these Pfam classifiers are too greedy. For example, the Pfam HMM Toxin_34 matches distant sequences we finally properly separate thanks to models SN_03_05, SN_04_00, SN_04_01, SN_04_02, SN_04_03, SN_04_04, SN_05_00, SN_25_00, and SN_31_00. Similarly, the Toxin_35 model from Pfam matches sequences finally distributed under 27 new classifiers from seven families (SN_02, SN_07, SN_09, SN_14, SN_19, SN_29, and SN_32). Therefore, using these Pfam classifiers for new peptides issued from transcriptome projects, and without any previous knowledge, could be completely confusing. The newly-proposed classifiers are better in most cases, even as global classifiers.

The ability of our profile HMMs to properly discriminate between closely-related sequence groups is illustrated with a deep analysis of the previous huwentoxin-1 family. Our analysis demonstrates that, for a given cysteine backbone (for example, C-C-CC-C-C for the huwentoxin-1 family), it is now possible to distinguish between peptides showing different numbers of amino acid residues between cysteines: for example, C-X(6)-C-X(5)-C-C-X(4)-C-X(8)-C for SN_10_01; C-X(6)-C-X(6)-C-C-X(4)-C-X(6)-C for SN_10_04, and C-X(6)-C-X(5)-C-C-X(4)-C-X(7)-C for SN_10_05 where X represents any amino acid. In addition, the new classifiers are sensitive to the chemical context in the sequence: for example, SN_10_22 represents a group of peptides having numerous polar uncharged residues around the double cysteine (S, N, Q) while, for peptides in the SN_10_13 class, the corresponding region is composed of hydrophobic (M, W) and charged (E) residues (Figure 1).

### 2.3. Hmmcompete, an Add-On to the HMMER3 Suite

**Figure 2:**
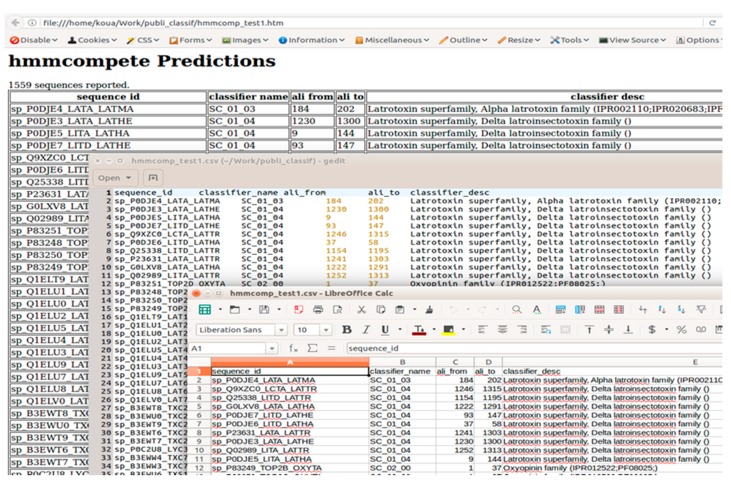
*hmmcompete* outputs. The standard output is a tab-delimited file that can be opened by text editors (Word, OOWriter, Wordpad, nano, vi, gedit, kate, etc.) or spreadsheet editors (Excel, OOCalc, etc.). An example of the tab-delimited output is provided as Supplementary File F5. An HTML can be saved with the option --htmout. An example of the HTML output is provided as Supplementary File F7.

The score obtained by a sequence for a given model reflects how closely the sequence is from the original alignment used to create the model. In addition, HMMs are predictive models able to identify remote homologous sequences. Consequently, building HMM classifiers for closely-related sequences necessarily implies closely-related models, which may match a single sequence during the *hmmsearch* process. This will result in a very large report file where every sequence may have several hits from these closely-related models showing closely-related bit scores. This situation can be annoying when a sequence database is searched using an HMM database. It cannot be avoided and requires a post-processing of the initial matching result. We, therefore, introduce the *hmmcompete* command line program as an add-on tool for the HMMER3 suite. *hmmcompete* is intended to monitor the classification realized by using a provided HMM dataset to match given peptide queries and to internally realize the post-processing step in order to provide only the best classification to the user. Protein query sequences must be given in FASTA format. *hmmcompete* produces modulable outputs users can visualize thanks to either text editors, spreadsheet software, or even web browsers. The possibility to display only desired columns in the result makes the tool handier (Figure 2).

#### 2.3.1. Synopsis

**hmmcompete** [options] --hmm <hmmDb> --in <seqFastaDb>

#### 2.3.2. Description

*hmmcompete* is proposed as an add-on to the HMMER3 suite. *hmmcompete* internally runs *hmmsearch* which is, therefore, required and available via the users’ PATH environment variable. *hmmcompete* is used to search a profile database against a sequence database. For each profile in <*hmmdb*>, that query is used to search the target database of sequences in <*seqFastaDb*>, and output for each sequence the best matching profile. *hmmcompete* is provided as an executable Perl script.

The standard output is a tab-separated file indicating for each sequence in <*seqFastaDb*> the model giving the best domain bit score and the corresponding domain position in the sequence. Bit scores for alternative matches can also be reported using the --altpred option. The -o option allows redirecting the main output, including throwing it away in /dev/null. <*hmmDB*> can be parsed on request (using the -d option) to extract and display profile HMM description lines. An HTML output can also be generated and saved for browser-mediated visualization (using the --htmout option). The --hsout option is also proposed for users interested in obtaining an *hmmsearch*-like output. All available options are listed in Table 4.

#### 2.3.3. Output Description

The result table associates one query sequence (from the *<seqFastaDb>* file) to its best match (from the <*hmmDb*>) (Figure 2, Supplementary Files F5 and F7). Sequences not matched by any provided model are not displayed by default. The option --allseq can be used to change this standard behavior and also display sequences not having any profile-based classification.

Each line consists of the following columns:

- *sequence_id*: the first part of target sequence FASTA header from the > sign to the first space.
- *classifier_name*: name of the profile giving the higher bit score for the considered peptide sequence.
- *ali_from*: the position in the target sequence at which the best hit starts.
- *ali_to*: the position in the target sequence at which the best hit ends.
- *target_region*: display region of the target sequence that matched the best model hit (region going from *ali_from* to *ali_to*). Only available if the --pepreg option is set.
- *classifier_desc*: description line (DESC) from the best classifier when available in the concerned HMM entry of the *<hmmDb>.* Only available if the --desc (-d) option is set.
- *matches_count*: number of models from the *<hmmDb>* producing a valuable match for the target peptide sequence. Only available if the --altpred option is set.
- *matches_position*: a list of all HMMs in *<hmmDb>* producing a match. Each hit is described by giving some useful details formatted as (ali_from-ali_to#classifier_name#bit_score). Only available if the --altpred option is set.

#### 2.3.4. hmmcompete Use Example

The overall classification of spider toxins from the ToxProt database has been realized using the new *hmmcompete* tool and the new spider-specific models using the following command:

hmmcompete --hmm ekenda_class.hmm -i ToxProt_reviewed.fas --allseq -d --pepreg -o ToxProt_reviewed_summarybyseq.csv

To be able to check the prediction given by our new models (ekenda_class.hmm) compared to ToxProt annotation, we used the -d option to include model descriptions in the output. These descriptions are inherited from the ToxProt current classification (Table 3). The “--allseq” option allowed easy checking of which sequences could not be classified by our models. The result file is provided as Supplementary File F9.

## 3. Discussion

### 3.1. Precision and Evolution of the Proposed Classification System

Analyses of newly-identified amino acid sequences from next-generation sequencing techniques should be done rapidly and should comprise general information about identified proteins concerning their precursors, structural domains, and a possible grouping into a given protein family. This is what our new classifiers are doing for spider venom components. Currently-available Pfam HMMs provide a one-level family classification where the toxin type has to be deduced from the profile documentation (or, hopefully, from the profile description line). For example, a peptide matched by the PF10530 (Toxin_35) HMM model is a spider neurotoxin with the ICK motif. More precise information cannot be obtained. From our analyses, Pfam classifiers appeared mainly to act as global classifiers matching numerous sequences sharing the same domain signature. Since the ICK motif is highly conserved in spider venom peptides, such a classifier can hide important sequence specificities. Based on a manual refinement of their underlying alignments, our new models represent human-guided machine learning classifiers. The main advantage of the newly-proposed models is the possibility to perform very sharp distinctions between closely-related peptides and reliably assign a sequence directly to its three classification levels with a single search (Figure 1 and Figure 2). Our models, therefore, allow to split old families containing peptides sharing a general feature (such as a given cysteine backbone) into refined groups characterized by a specific amino acid context, a specific sequence length, or a more precise amino acid conservation state. The example of the previous huwentoxin-1 clearly demonstrates this ability. Our models, therefore, represent a valuable improvement for the classification of spider venom components. In this transcriptomic era, such classifiers will allow a speedier identification and classification of interesting peptides from venom gland transcriptomic projects.

For naming families and related sequence groups in the proposed classification system, we decided to follow a neutral numerical naming. Numerical classification systems are common in biology and have, for example, been adopted by Pfam (profile HMMs used to classify spider toxins in ToxProt are named following an ordinal system: Toxin_12 to Toxin_35), InterPro, Gene Ontology, and the Enzyme Commission, etc. The advantage of our numerical system is to distinguish venom peptide class names from their taxonomic family names. This will greatly reduce the confusion introduced by historical names. On the other hand, creating specific models for spiders allows avoiding transferring mammalian domain names into spider peptide and protein classification. Another important advantage of the adopted numerical system is its robust evolution potentiality. Indeed, if a new neurotoxin sequence is identified and does not match any existing classifier, a new spider neurotoxin family can be created using the next level 2 ordinal value, without any need to rename existing families. Similarly, if a structurally-different group of toxin is identified in an existing family, this group will simply receive the next ordinal value for level 3.

Adopting a complete numerical system may hide meaningful relationships venom components might have (for example, evolutionary, biophysical, and pharmacological relationships). Indeed, using names indicating a pharmacological activity could facilitate further analysis of classified peptides. However, when analyzing datasets of peptides sharing the same pharmacological activity, we realized that these peptides present different cysteine distributions and inter-cysteine structures (Supplementary Files F4–F7). This makes it difficult to design a valuable classifier for pharmacological activity annotation and, therefore, name a model based on pharmacological function. Finally, considering that a machine-based classification is merely indicative, and to avoid researchers to wrongly assume the effective biological activity of peptides based on their proposed HMM-based classification, the neutral family naming was preferred. However, to facilitate the transition between commonly-used names and the system proposed here, previous ToxProt annotation was added as a description of the new profile HMMs (DESC field, Supplementary File F2). This description can be displayed in the classification result performed using both the new classifier and *hmmcompete* with the option “--desc” or “-d”. In any case, the structural relationship remains the basis of group formation in the new system. Additional relationships could still be obtained from sequences names.

### 3.2. hmmcompete Allows Taking Advantage of New Classifiers

When constructing classifiers for closely-related sequence groups, we were aware about the risk of obtaining multiple profile HMM matches for a single peptide sequence. Profile HMMs are normally intended to represent the maximum sequence heterogeneity and are, therefore, used to detect remote homologous sequences. For example, the Pfam Toxin_35 model has been built with nine sequences, but is currently fishing 171 reviewed sequences from ToxProt. Trying to achieve a sharp classification using pHMMs was, therefore, a challenging initiative. Many annotation strategies take advantage of the competition between classifiers to provide fine classification [11,15,16]. However, to our knowledge, *hmmcompete* is the first open standalone HMMER3-compatible tool to realize high-throughput sharp classification based on competition between profile HMMs.

From a technical point of view, *hmmcompete* is a Perl script. However, *hmmcompete* is not based on BioPerl, which also implements an *hmmsearch* result parser. Such a choice would have implied either distributing *hmmcompete* along with BioPerl or to require HMMER3 users to install this package. We adopted a simpler solution and proposed a Perl script that one can place in the same directory of *hmmsearch* and save a full BioPerl installation because of a single parser. Therefore, we wrote a new parser for *hmmsearh* table output instead of re-implementing the internal object-oriented data structure of BioPerl. The HMMER3 users guide explicitly indicates to avoid parsing the --domtblout output of *hmmsearch*. This is, first, because this output is space-delimited rather than tab-delimited and, second, because some columns like target name (column 1) and query name (column 4) contain a text value that may accidentally have space characters. However, since this output is the most accessible from *hmmsearch*, we decided to take the risk to write a dedicated parser based on it. This risk is minimized by two major precautions. First, the protein sequence dataset must be in FASTA format. This precaution ensures that the target sequence name does not contain any space character. We take advantage of the fact that *hmmsearch* internally truncates the sequence name by taking the section between the > sign and the first space character. Secondly, as we did for our new profile HMMs, space characters are generally avoided in profile HMM names. Therefore, our *hmmcompete* program could be safely used, provided that the space character is avoided for both sequence and profile names.

## 4. Conclusions

The number of spider venom-derived sequences needed to be properly classified is constantly increasing, especially due to next-generation transcriptomic studies. We have constructed 219 profile HMMs able to reliably classify these sequences using a new system that comprises three classification levels: the sequence type, the sequence family, and groups inside these families. In addition to this new classifier set, we introduce a new bioinformatics tool to the HMMER3 toolkit. This new tool is based on *hmmsearch* and is intended to simplify users’ classification work. Even if *hmmcompete* is proposed as a command line tool, its tabular output could be viewed using both Excel-like or text editing software, making the classification easy to handle by any end-user. This program, as well as the newly-designed pHMM classifiers, constitute promising tools for functional analyses of spider venom components. The HMM-based strategy proposed in this study can be used to improve the classification of other spider peptides (antimicrobial peptides derived from spider hemocytes) as well as venom peptides from other animal groups (snakes, cone snails, scorpiones, anemones, etc.). One should, however, produce a dedicated set of profile HMMs. The *hmmcompete* script can be used and integrated in any annotation pipeline and will manage all HMMER 3 profiles.

## 5. Materials and Methods

### 5.1. Data Acquisition

Data used in this study were extracted from UniProtKB release 2017_04 (on 20 April) using the following search expression: *taxonomy:"Araneae (spiders) [6893]" keyword:"Toxin [KW-0800]" AND reviewed:yes*. A total of 1379 sequences manually reviewed by the ToxProt project were extracted. The manual annotation provided by UniProtKB was used as a reference for quality control for the newly-built profiles. Toxin sequences were extracted along with InterPro signatures (43) and Pfam identifiers (24 from the ToxProt export, but only 23 were used, the HMM of conotoxin was not considered) were used as current associated classifiers for comparison with our newly-designed classifiers. Pfam HMMs are downloaded from the Pfam server for comparison purposes. Further spider venom proteins and possible toxic peptides from our own transcriptomes and genebank data were manually annotated and analyzed concerning possible InterPro signatures.

### 5.2. Model Construction

Peptide classification in this study was made by using profile HMMs that have been shown to be excellent classifiers even for closely related peptides [15,16,17,18].

As indicated previously, the ToxProt family organization of spider toxins is a two-level classification system. Occasionally the first classification level is referred to as the superfamily and, in this case, the second classification level is considered a family. In other cases, the first classification level is referred to as a family, making the second level a subfamily. In this study, we simply consider ToxProt-level1 (TPL1) and ToxProt-level2 (TPL2) to refer to classification levels found in the ToxProt annotation. Data extracted from ToxProt were distributed in individual files based on their TPL1 and TPL2 annotation. A file related to a TPL1 classification contained all peptides related to that level in addition to all TPL2 peptides associated to the given TPL1. A TPL2 file only contained peptides related to that specific level. In addition, sequences were divided into structural conservation groups based on (i) the distribution of cysteine residues; (ii) on the number of amino acid between conserved cysteine; and (iii) on amino acid properties (charge, hydrophobicity, size).

Individual multiple sequence alignments (MSA) were made for each resulting sequence file using the *linsi* program (version v7.273 (2016/2/20) from the MAFFT suite [19]. This resulted in a total of 233 MSAs that were manually checked. These MSAs were manually processed using the Jalview program (version 2.9.0b2) [20] to trim alignments to the core cysteine motif, remove fragments, and to only keep unique, mature peptides. According to sequence similarity, and in order to improve the final alignment, some files were split again. This resulted in the production of 247 cleaned MSAs.

Cleaned MSAs were then used to build new profile HMMs using the *hmmbuild* program from the HMMER3 software suite (version 3.1b2). For each toxin family, our profile HMM construction methodology included a validation step where profile HMMs’ predictive performance was assessed by testing either with true positive and false positive datasets, as well as on random sequences using the *hmmsearch* program.

### 5.3. Profile HMM-Based Classification

In-house scripts were developed for prediction assignment when multiple profile HMMs matched a given peptide. A sequence was predicted to belong to the family whose profile produced the higher score. The domain bit scores (column 14 of the tabular output of *hmmsearch* with the option *--domtblout*) were used for family assignment. These scores do not depend on the size of the sequence database, but only on the profile HMM and the target sequence. They correspond to the single best-scoring domain in the sequence, rather than the sum of all its identified domains (HMMER3 user guide). In this study, the goal was to properly classify and discriminate closely-related peptides. In addition, profile HMMs were built for the core domain of every classification level. We, therefore, considered the best domain score as the best criteria to assign a peptide to a given classification group.

## Figures and Tables

**Table 1:** InterPro signatures and Pfam HMMs used for spider toxin classification and related peptide family names used in ToxProt (as of 20 April 2017).

InterPro Signature Combination	Pfam HMM	ToxProt First Classification Level (TPL1) *	Total of Annotated Sequences in ToxProt
IPR000737; IPR011052	PF00299	Protease inhibitor I7 (squash-type serine protease inhibitor) family	1
IPR002110; IPR020683	PF00023; PF12796	Latrotoxin superfamily	4
IPR002110; IPR020683; IPR013829	PF00023; PF12796; PF13606	Latrotoxin superfamily	3
IPR002223	PF00014	Venom Kunitz-type family	39
IPR002223; IPR020901	PF00014	Venom Kunitz-type family	8
IPR003614			1
IPR004169		Plectoxin superfamily (16)	18
		Spider toxin CSTX superfamily (1)	
		No class (1)	
IPR004214	PF02950	Spider toxin Tx2 family (1)	2
		Huwentoxin-1 family (1)	
IPR005853; IPR013605	PF08396	Omega-agatoxin superfamily	13
IPR007733; IPR027300	PF05039	Spider agouti family	1
IPR008017	PF05353	Delta-atracotoxin family	7
IPR008197		Spider wap-1 family(17)	21
		Spider wap-2 family (4)	
IPR009243		Beta/delta-agatoxin family	12
IPR009243; IPR004169		Beta/delta-agatoxin family	2
IPR009415	PF06357	Shiva superfamily	13
IPR009415; IPR018071	PF06357	Shiva superfamily (14)	15
		No class (1)	
IPR011142		Spider toxin CSTX superfamily	6
IPR011696	PF07740	Huwentoxin-1 family	114
IPR011696; IPR013140	PF07740	Huwentoxin-1 family	119
IPR011696; IPR016191	PF07740	Huwentoxin-1 family	4
IPR012499	PF07945	Shiva superfamily	7
IPR012522	PF08025	Oxyopinin-2 family	4
IPR012625	PF08089	Huwentoxin-2 family	79
IPR012625; IPR012627	PF08092	Magi-1 superfamily	1
IPR012626	PF08091	Insecticidal toxin ABC family	5
IPR012627	PF08092	Magi-1 superfamily	82
IPR012628	PF08093	Magi-5 family	3
IPR012633	PF08115	Spider toxin SFI family	10
IPR012634	PF08116	Spider neurotoxin 21C2 family	4
IPR013139; IPR012628	PF08093	Omega-atracotoxin type 2 family	5
IPR013605	PF08396	Omega-agatoxin superfamily	14
IPR016328; IPR009243		Beta/delta-agatoxin family	13
IPR017946		Arthropod phospholipase D family	199
IPR017946; IPR000909		Arthropod phospholipase D family	2
IPR018802	PF10279	Latarcin superfamily	11
IPR019553	PF10530	Plectoxin superfamily (1)	62
		Spider toxin CSTX superfamily (6)	
		U6-lycotoxin family(10)	
		U7-lycotoxin family (11)	
		U8-lycotoxin family (28)	
		U11-lycotoxin family (6)	
IPR019553; IPR004169	PF10530	U10-lycotoxin family	5
IPR019553; IPR011142	PF10530	Spider toxin CSTX superfamily	104
IPR020683		Latrotoxin superfamily	1
IPR020683; IPR007094		Latrotoxin superfamily	1
IPR023569	PF06607	AVIT (prokineticin) family	9
IPR024079; IPR001506; IPR006026	PF01400	Peptidase M12A family	1
IPR027300		Plectoxin superfamily (5)	6
		No class (1)	
IPR027300; IPR004169		No class	1
IPR034035; IPR024079; IPR001506; IPR006026	PF01400	Peptidase M12A family	4

* When more than one family name is associated to a given signature, the number of sequences annotated as a member of each family is indicated between parentheses.

**Table 2:** Distribution of spider toxin sequences from ToxProt not associated to any InterPro signature or to any peptide family (25% of the sequences).

ToxProt Family	Number of Sequences
Aptotoxin family	4
Arthropod phospholipase D family	13
AVIT (prokineticin) family	1
Bradykinin-related peptide family	5
Cupiennin family	43
Cytoinsectotoxin family	20
Helical arthropod-neuropeptide-derived (HAND) family	3
Huwentoxin-1 family	12
HWTX-LSTX family	2
Insecticidal toxin DTX family	3
JZTX-72 family	3
Latrotoxin superfamily	1
Litx family	3
Magi-1 superfamily	1
Omega-agatoxin superfamily	2
Omega-lycotoxin family	7
Phrixotoxin family	13
Plectoxin superfamily	7
Shiva superfamily	2
Spider agouti family	9
Spider LiTx3-related peptide family	2
Spider toxin CSTX superfamily	6
Spider toxin Tx2 family	7
Spider toxin Tx3-6 family	7
Spider wap-1 family	1
U12-lycotoxin family	6
U2-agatoxin family	24
Venom metalloproteinase (M12B) family	1
No family name	114

A total of 229 peptides (17%) from the database are classified, but not related to any InterPro signature. A total of 114 sequences (8%) from the database are associated to neither an InterPro signature nor a similarity-based classification. This situation indicates that more classifiers are needed and/or the sensitivity of current classifiers has to be improved.

**Table 3:** Distribution of the new spider toxin profile HMMs and their related InterPro and Pfam signatures.

Toxin Type (Level 1)	Classifiers * (Level 2 and 3)	Discriminative ToxProt Annotation	InterPro Signatures	Pfam HMMs
Spider Cationic peptides (SC) 21 profile HMMs	SC_01_00	Cytoinsectotoxin family		
	SC_02_00	Oxyopinin family	IPR012522	PF08025
	SC_03_00 to SC_03_07	Latarcin superfamily	IPR018802	PF10279
	SC_04_01 to SC_04_10	Cupiennin family		
		CsTx-16 **		
	SC_05_00	Bradykinin-related peptide family		
Spider Neurotoxin (SN) 170 profile HMM	SN_01_00	U2-agatoxin family		
	SN_02_00 to SN_02_09	Plectoxin superfamily	IPR004169	
		CsTx-19 **, CsTx-28,34,36 ***		
	SN_03_01 to SN_03_06	Spider toxin Tx2 family	IPR004214	PF02950
	SN_04_00 to SN_04_04	Omega-agatoxin superfamily	IPR005853; IPR013605	PF08396
	SN_05_00 to SN_05_06	Spider agouti family	IPR007733; IPR027300	PF05039
	SN_06_00	Delta-atracotoxin family	IPR008017	PF05353
	SN_07_00 to SN_07_04	Beta/delta agatoxin family	IPR009243	
	SN_08_01 to SN_08_02	Shiva superfamily, Omega-toxin family	IPR009415; IPR018071	PF06357
	SN_09_00	Spider toxin Tx3-6 family		
	SN_10_00 to SN_10_67	Huwentoxin-1 family	IPR011696	PF07740
	SN_11_00	Shiva superfamily, Kappa toxin family	IPR012499	PF07945
	SN_12_01 to SN_12_08	Huwentoxin-2 family	IPR012625	PF08089
	SN_13_00 to SN_13_03	Insecticidal toxin ABC family	IPR012626	PF08091
	SN_14_00 to SN_14_09	Magi-1 superfamily	IPR012627	PF08092
	SN_15_01 to SN_15_02	Magi-5 family	IPR012628	PF08093
	SN_16_00	Spider toxin SFI family	IPR012633	PF08115
	SN_17_00	Spider neurotoxin 21C2 family	IPR012634	PF08116
	SN_18_00	AVIT (prokineticin) family	IPR023569	PF06607
	SN_19_00 to SN_19_12	Spider toxin CsTx superfamily	IPR019553; IPR011142	PF10530
	SN_20_00	CsTx-20 **		
	SN_21_00	Aptotoxin_family		
	SN_22_00	Helical arthropod neuropeptide derived (HAND) family		
	SN_23_00	Double-knot toxin subfamily		
	SN_24_00	OAIP 4 subfamily		
	SN_25_00	HWTX-LSTX family		
	SN_26_00	Insecticidal toxin DTX family		
	SN_27_00	JZTX-72 family		
	SN_28_00	Litx family		
	SN_29_00	Omega lycotoxin family		
	SN_30_00	Phrixotoxin family		
	SN_31_00	U12-lycotoxin family		
	SN_32_00 to SN_32_02	MIT-like AcTx family **	IPR020202	PF17556
		CsTx-21 **, CsTx-22 ***		
	SN_33_00	CsTx-26 ***		
	SN_34_00	CsTx-29 ***		
	SN_35_00	CsTx-35 ***		
	SN_36_00	Huwentoxin type 10 **		
	SN_37_00	CsTx-37 **		
	SN_38_00	CsTx-38 **		
	SN_39_00	Spider LiTx3 related peptide family		
	SN_40_00	Spiderine **		
Venom Proteins (VP) 28 profile HMMs	VP_01_00	Protease inhibitor I7 (squash type serine protease inhibitor) family	IPR000737; IPR011052	PF00299
	VP_02_00	Peptidase M12A family	IPR024079; IPR001506;	PF01400
	VP_03_01 to VP_03_02	Arthropod phospholipase D family	IPR017946	
	VP_04_00	Venom metalloproteinase (M12B) family		
	VP_05_00	Hyaluronidase **	IPR018155	
	VP_06_00	Arthropod Phospholipase A2 **	IPR001211	
	VP_07_00	Angiotensin-converting Enzyme **	IPR033591	
	VP_08_00	Peptidylglycine alpha-amidating monooxygenase **	IPR000720	
	VP_09_00	Signal peptidase **	IPR001733	
	VP_10_00	Venom serine protease ***	IPR001314	
	VP_11_01 to VP_11_02	Spider WAP family	IPR008197	
	VP_12_01 to VP_12_04	Venom Kunitz-type family	IPR002223; IPR020901	PF00014
	VP_13_01 to VP_13_02	Cysteine-rich secretory protein	IPR014044;	
			IPR002413	
	VP_14_00	Thyroglobulin-like protein **	IPR000716	
	VP_15_00	Leucine rich peptide **	IPR032675	
	VP_16_00	Protein disulfide-isomerase **	IPR005792	
	VP_17_00	Tachylectin 5A **	IPR002181	
	VP_18_00	Cystatin **	IPR027214	
	VP_19_01 to VP_19_04	Latrotoxin superfamily	IPR002110; IPR020683	PF00023

*: When the level 2 classifier concerns very divergent sequences, the family profile (xx_nn_00) showed poor classification performance and were, therefore, eliminated. **: Sequences from these families were not present in ToxProt. The family is documented in UniProtKB (Venomzone). ***: These families are newly detected or in unpublished venom gland transcriptomes. The new proposed classifiers are not only intended to reorganize the ToxProt classification, but improve the overall coverage for all types of known venom components.

**Table 4:** Complete list of *hmmcompete* options.

Option Name	Description
--hmm <hmmDbPath>	The profile database to be used for sequence classification. HMMER3 profiles. This is a mandatory argument.
-i or --in <seqFastaDb>	The sequence database to be classified. In FASTA format. This is a mandatory argument.
-h or --help	Help. Print a brief reminder of command line usage and available options. *hmmcompete* help page will also be displayed if the command is executed without any argument.
-v or --version	Print *hmmcompete* version and exit.
-o <file_path> or --out <file_path>	Direct the main tabular output to a file <f> instead of the default stdout.
-d or --desc	Display profile description in the main output when the description is present in the profile. Default: ‘Off’.
--altpred	Display number of alternative profile HMM matching a sequence, as well as a summarized description of each alternative match. This description includes positions of the query sequence matching the profile, as well as the produced score. Default: ‘Off’.
--allseq	Also report sequences not matched by any model. Default: Off, i.e., only query sequences matched by a profile in hmmDB are reported by default.
--pepreg	Display region of the target sequence that matched the reported profileHMM. Default: ‘Off’.
--hsout <file_path>	Save an output file similar to that of *hmmsearch* with the --domtblout option. Will only report the best prediction/classification where available. Sequences not matched by any model are not reported. Alternative profile HMM matches are also ignored.
--htmout <file_path>	Save an HTML version of the output. May be useful for web integration.

## Supplementary Materials

The following are available online at www.mdpi.com/2072-6651/9/8/245/s1, **File F1**. ekenda_class.hmm is a text file containing the 219 new proposed profile HMMs. **File F2**. classif_level.xls is a detailed list with the ToxProt family corresponding to each new profile HMM. **File F3**. *hmmcompete* is an executable Perl file to be placed in the same directory as *hmmsearch* and made accessible in the PATH environment variable. **File F4**. mu_neurotoxin.fas is an annotated fasta file of spider mu-neurotoxins provided as test set. **File F5**. mu_neurotoxin_classif.csv is an example of *hmmcompete* tab-delimited output file obtained by classifying File F4 with the new classifiers (by running the command: hmmcompete --hmm ekenda_class.hmm -i mu_neurotoxin.fas --allseq -d --hmmaln --inanot -o mu_neurotoxin_classif.csv). **File F6**. omega_neurotox.fas is an annotated fasta file of spider omega-neurotoxins provided as test set. **File F7**. omega_neurotoxin_classif.htm is an example of *hmmcompete* web page output file obtained by classifying File F6 with the new classifiers (by running the command: hmmcompete --hmm ekenda_class.hmm -i omega_neurotoxins.fasta --allseq -d --pepreg --inanot --htmout omega_neurotoxin_classif.htm -o/dev/null). **File F8**. ToxProt_classif_comparison.csv is the output of the global comparison between the proposed models and Pfam classifiers for classification of ToxProt reviewed entries. **File F9**. ToxProt_reviewed_summarybyseq.csv is the output of the classification of all ToxProt reviewed entries using the proposed profile HMMs.
