# Supplementary material for: Spider Neurotoxins, Short Linear Cationic Peptides and Venom Protein Classification Improved by an Automated Competition between Exhaustive Profile HMM Classifiers
Source: Toxins (Basel). 2017 Aug 8;9(8):245. doi: 10.3390/toxins9080245 (PMC5577579; doi:10.3390/toxins9080245)
Supplement: Supplementary file 1 [file toxins-09-00245-s001.zip › omega_neurotoxin_classif.htm]

# hmmcompete Result

Predictions based on ekenda\_class.hmm

117 sequences reported.  

sequence id | seq annotation | classifier name | ali from | ali to | target region | classifier desc || sp|P83911|TX37\_PHORI | Omega-ctenitoxin-Pr1a | SN\_02\_00 | 2 | 33 | CAGLYKKCGKGVNTCCENRPCKCDLAMGNCIC | Plectoxin superfamily (IPR004169;) |
| sp|P0DL48|TX3\_AGEAP | Omega-agatoxin-Aa5a | SN\_02\_00 | 3 | 32 | CAGAYKSCDKVKCCHDRRCRCNIAMDNCVC | Plectoxin superfamily (IPR004169;) |
| sp|O76201|TX32\_PHONI | Omega-ctenitoxin-Pn1a | SN\_02\_00 | 39 | 70 | CAGLYKKCGKGASPCCEDRPCKCDLAMGNCIC | Plectoxin superfamily (IPR004169;) |
| sp|P81789|TX33\_PHONI | Omega-ctenitoxin-Pn2a (Fragment) | SN\_02\_01 | 2 | 32 | CANAYKSCNGPHTCCWGYNGYKKACICSGXN | Plectoxin superfamily, Tx3 family (IPR004169;) |
| sp|P34079|TXPL2\_PLETR | Omega-plectoxin-Pt1a | SN\_02\_02 | 7 | 40 | GDTCDHTKKCCDDCYTCRCGTPWGANCRCDYYKA | Plectoxin superfamily, Plectoxin family (IPR004169;) |
| sp|P30288|TOG4A\_AGEAP | Omega-agatoxin-Aa4a | SN\_02\_03 | 4 | 36 | CIAKDYGRCKWGGTPCCRGRGCICSIMGTNCEC | Plectoxin superfamily, Type IV omega agatoxin family (IPR004169;) |
| sp|P37045|TOG4B\_AGEAP | Omega-agatoxin-Aa4b | SN\_02\_03 | 39 | 71 | CIAEDYGKCTWGGTKCCRGRPCRCSMIGTNCEC | Plectoxin superfamily, Type IV omega agatoxin family (IPR004169;) |
| sp|P81790|TX34\_PHONI | Omega-ctenitoxin-Pn3a | SN\_04\_00 | 41 | 111 | CINVGDFCDGKKDDCQCCRDNAFCSCSVIFGYKTNCRCEVGTTATSYGICMAKHKCGRQTTCTKPCLSKRC | Omega-agatoxin superfamily (IPR005853;IPR013605;PF08396;) |
| sp|P15969|TOG1A\_AGEAP | Omega-agatoxin-1A | SN\_04\_01 | 46 | 92 | CDGNESDCKCYGKWHKCRCPWKWHFTGEGPCTCEKGMKHTCITKLHC | Omega-agatoxin superfamily, Omega agatx sb2 (IPR005853;IPR013605;PF08396;) |
| sp|P15970|TOG1B\_AGEAP | Omega-agatoxin-Aa1b (Fragment) | SN\_04\_01 | 10 | 35 | CDGNESDCKCAGAWIKCRCPPMWHIN | Omega-agatoxin superfamily, Omega agatx sb2 (IPR005853;IPR013605;PF08396;) |
| sp|P15971|TOG2A\_AGEAP | Omega-agatoxin-Aa2a (Fragment) | SN\_04\_03 | 2 | 28 | CIEIGGDCDGYQEKSYCQCCRNNGFCS | Omega-agatoxin superfamily, Type II III omega agatoxin family (IPR005853;IPR013605;PF08396;) |
| sp|P33034|TOG3A\_AGEAP | Omega-agatoxin-Aa3a | SN\_04\_04 | 2 | 67 | CIDIGGDCDGEKDDCQCCRRNGYCSCYSLFGYLKSGCKCVVGTSAEFQGICRRKARQCYNSDPDKC | Omega-agatoxin superfamily, omega agatox sb3 (IPR005853;IPR013605;PF08396;) |
| sp|P81744|TOG3B\_AGEAP | Omega-agatoxin-Aa3b | SN\_04\_04 | 2 | 67 | CIDFGGDCDGEKDDCQCCRSNGYCSCYNLFGYLKSGCKCEVGTSAEFRRICRRKAKQCYNSDPDKC | Omega-agatoxin superfamily, omega agatox sb3 (IPR005853;IPR013605;PF08396;) |
| sp|P81745|TOG3C\_AGEAP | Omega-agatoxin-Aa3c (Fragment) | SN\_04\_04 | 2 | 43 | CIDFGGDCDGEKDDCQCCXRNGYCSCYNLFGYLKRGCKXEVG | Omega-agatoxin superfamily, omega agatox sb3 (IPR005853;IPR013605;PF08396;) |
| sp|P81746|TOG3D\_AGEAP | Omega-agatoxin-Aa3d (Fragment) | SN\_04\_04 | 2 | 37 | CIKIGEDCDGDKDDCQCCRTNGYCSXYXLFGYLKSG | Omega-agatoxin superfamily, omega agatox sb3 (IPR005853;IPR013605;PF08396;) |
| tr|A0A0G3F8Z3|A0A0G3F8Z3\_9ARAC | Omega-Tbo-IT1 | SN\_07\_01 | 39 | 69 | CASKNERCGNALYGTKGPGCCNGKCICRTVP | Beta-delta agatoxin family, Aga 2 subfamily (IPR009243;) |
| sp|P0DMQ2|TO1C\_HADMO | Omega-hexatoxin-Hmo1c | SN\_08\_01 | 51 | 83 | CTRTDQPCPYDQDCCSGSCTLKKNENGNLVKRC | Shiva Omega superfamily, Omega toxin family (IPR009415;PF06357;) |
| tr|A0A1D0C0X6|A0A1D0C0X6\_HADIN | Omega-hexatoxin-Hi1g\_6 | SN\_08\_01 | 45 | 77 | CTPTDQPCPYDESCCSGSCTYKANENGNQVKRC | Shiva Omega superfamily, Omega toxin family (IPR009415;PF06357;) |
| tr|S0F205|S0F205\_HADVN | Omega-hexatoxin-Hvn1b\_2 insecticidal toxin | SN\_08\_01 | 51 | 83 | CIPSGQPCPYNENCCSKSCTYKENENGNTVQRC | Shiva Omega superfamily, Omega toxin family (IPR009415;PF06357;) |
| sp|P83580|TO1A\_ATRRO | Omega-hexatoxin-Ar1a | SN\_08\_01 | 52 | 84 | CIPSGQPCPYNEHCCSGSCTYKENENGNTVQRC | Shiva Omega superfamily, Omega toxin family (IPR009415;PF06357;) |
| sp|A5A3H1|TO1B\_ATRRO | Omega-hexatoxin-Ar1b | SN\_08\_01 | 40 | 72 | CTPTGQPCPYNESCCSGSCQEQLNENGHTVKRC | Shiva Omega superfamily, Omega toxin family (IPR009415;PF06357;) |
| tr|A0A1D0BPR0|A0A1D0BPR0\_HADIN | Omega-hexatoxin-Hi1a\_8 | SN\_08\_01 | 45 | 67 | CTPTDQPCPYHESCCSGSCTYKA | Shiva Omega superfamily, Omega toxin family (IPR009415;PF06357;) |
| tr|A0A1D0BND6|A0A1D0BND6\_HADIN | Omega-hexatoxin-Hi1a\_6 | SN\_08\_01 | 45 | 77 | CTPTDQPCPYHESCCSGSCTYKANENGNQVKRC | Shiva Omega superfamily, Omega toxin family (IPR009415;PF06357;) |
| sp|P81599|TO1F\_HADVE | Omega-hexatoxin-Hv1f | SN\_08\_01 | 4 | 36 | CIPSGQPCPYSKYCCSGSCTYKTNENGNSVQRC | Shiva Omega superfamily, Omega toxin family (IPR009415;PF06357;) |
| sp|P0C2L7|TO1C\_HADIN | Omega-hexatoxin-Hi1c | SN\_08\_01 | 4 | 36 | CIRTDQPCPYNESCCSGSCTYKANENGNQVKRC | Shiva Omega superfamily, Omega toxin family (IPR009415;PF06357;) |
| sp|S0F1M4|TO1A\_HADVN | Omega-hexatoxin-Hvn1a | SN\_08\_01 | 50 | 82 | CIPSGQPCPYNENCCSKSCTYKEMKTATPVQRC | Shiva Omega superfamily, Omega toxin family (IPR009415;PF06357;) |
| sp|S0F1N0|TO1E\_HADIN | Omega-hexatoxin-Hi1e | SN\_08\_01 | 46 | 78 | CIPTGQPCPYNENCCSQSCTYKANENGNQVKGC | Shiva Omega superfamily, Omega toxin family (IPR009415;PF06357;) |
| sp|P81597|TO1D\_HADVE | Omega-hexatoxin-Hv1d | SN\_08\_01 | 4 | 36 | CIPSGQPCPYNENCCSKSCTYKENENGNTVQRC | Shiva Omega superfamily, Omega toxin family (IPR009415;PF06357;) |
| tr|A0A1D0BZY4|A0A1D0BZY4\_HADIN | Omega-hexatoxin-Hi1b\_11 | SN\_08\_01 | 46 | 78 | CIPTGQPCPYNENCCSQSCTYKANENGNQVKRC | Shiva Omega superfamily, Omega toxin family (IPR009415;PF06357;) |
| tr|S0F1M8|S0F1M8\_HADIN | Omega-hexatoxin-Hi1g\_2 insecticidal toxin | SN\_08\_01 | 45 | 77 | CTPTDQPCPYDESCCSGSCTYKANENGNQVKRC | Shiva Omega superfamily, Omega toxin family (IPR009415;PF06357;) |
| tr|A0A1D0BRC8|A0A1D0BRC8\_HADIN | Omega-hexatoxin-Hi1h\_1 | SN\_08\_01 | 45 | 77 | CTPTNQPCPYHESCCSGSCTYKANENGNQVKRC | Shiva Omega superfamily, Omega toxin family (IPR009415;PF06357;) |
| tr|A0A1D0BNE8|A0A1D0BNE8\_HADIN | Omega-hexatoxin-Hi1a\_10 | SN\_08\_01 | 70 | 102 | CTPTDQPCPYHESCCSGSCTYKANENGNQVKRC | Shiva Omega superfamily, Omega toxin family (IPR009415;PF06357;) |
| sp|P0DMQ0|TO1A\_HADMO | Omega-hexatoxin-Hmo1a | SN\_08\_01 | 51 | 83 | CTRTDQPCPYNEDCCSGSCTLKKNENGNLVKRC | Shiva Omega superfamily, Omega toxin family (IPR009415;PF06357;) |
| sp|A5A3H5|TO1F\_ATRRO | Omega-hexatoxin-Ar1f | SN\_08\_01 | 45 | 77 | CIPSGQPCPYNENCCSQSCTFKENETGNTVKRC | Shiva Omega superfamily, Omega toxin family (IPR009415;PF06357;) |
| tr|A0A1D0C034|A0A1D0C034\_HADIN | Omega-hexatoxin-Hi1i\_1 | SN\_08\_01 | 45 | 77 | CTPTDQPCPYNESCCSGSCTYKANENGNQVKRC | Shiva Omega superfamily, Omega toxin family (IPR009415;PF06357;) |
| sp|S0F204|TO1F\_HADIN | Omega-hexatoxin-Hi1f | SN\_08\_01 | 46 | 78 | CIPTGQPCPYNENCCSQSCTYKTNENGNQVKGC | Shiva Omega superfamily, Omega toxin family (IPR009415;PF06357;) |
| sp|S0F215|TO1G\_HADIN | Omega-hexatoxin-Hi1g | SN\_08\_01 | 45 | 77 | CTPTDQPCPYDESCCSGSCTYKANENGNQVKRC | Shiva Omega superfamily, Omega toxin family (IPR009415;PF06357;) |
| tr|A0A1D0C045|A0A1D0C045\_HADIN | Omega-hexatoxin-Hi1g\_5 | SN\_08\_01 | 45 | 77 | CTPTDQPCPYDESCCSGSCTYKANENGNQVKRC | Shiva Omega superfamily, Omega toxin family (IPR009415;PF06357;) |
| sp|P56207|TO1A\_HADVE | Omega-hexatoxin-Hv1a | SN\_08\_01 | 4 | 36 | CIPSGQPCPYNENCCSQSCTFKENENGNTVKRC | Shiva Omega superfamily, Omega toxin family (IPR009415;PF06357;) |
| tr|S0F212|S0F212\_HADVN | Omega-hexatoxin-Hvn1b\_6 insecticidal toxin | SN\_08\_01 | 51 | 83 | CIPSGQPCPYNENCCSKSCTYKENENGNTVQRC | Shiva Omega superfamily, Omega toxin family (IPR009415;PF06357;) |
| sp|A5A3H2|TO1C\_ATRRO | Omega-hexatoxin-Ar1c | SN\_08\_01 | 45 | 77 | CIPSGQPCPYNENYCSQSCTFKENENANTVKRC | Shiva Omega superfamily, Omega toxin family (IPR009415;PF06357;) |
| tr|S0F1N4|S0F1N4\_HADIN | Omega-hexatoxin-Hi1g\_1 insecticidal toxin | SN\_08\_01 | 45 | 77 | CTPTDQPCPYDESCCSGSCTYKANENGNQVKRC | Shiva Omega superfamily, Omega toxin family (IPR009415;PF06357;) |
| sp|P81595|TO1B\_HADVE | Omega-hexatoxin-Hv1b | SN\_08\_01 | 4 | 36 | CIPSGQPCPYNENCCSQSCTYKENENGNTVKRC | Shiva Omega superfamily, Omega toxin family (IPR009415;PF06357;) |
| sp|P81598|TO1E\_HADVE | Omega-hexatoxin-Hv1e | SN\_08\_01 | 4 | 36 | CIPSGQPCPYNENCCSQSCTYKENENGNTVKRC | Shiva Omega superfamily, Omega toxin family (IPR009415;PF06357;) |
| sp|S0F1N7|TO1B\_HADVN | Omega-hexatoxin-Hvn1b | SN\_08\_01 | 51 | 83 | CIPSGQPCPYNENCCSKSCTYKENENGNTVQRC | Shiva Omega superfamily, Omega toxin family (IPR009415;PF06357;) |
| sp|P0C2L4|TO1B\_HADFO | Omega-hexatoxin-Hf1a | SN\_08\_01 | 4 | 36 | CIRSGQPCPYNENCCSQSCTFKTNENGNTVKRC | Shiva Omega superfamily, Omega toxin family (IPR009415;PF06357;) |
| tr|A0A1D0C0Y4|A0A1D0C0Y4\_HADIN | Omega-hexatoxin-Hi1a\_9 | SN\_08\_01 | 45 | 77 | CTPTDQPCPYHESCCSGSCTYKANENGNQVKRC | Shiva Omega superfamily, Omega toxin family (IPR009415;PF06357;) |
| sp|P0DMQ1|TO1B\_HADMO | Omega-hexatoxin-Hmo1b | SN\_08\_01 | 51 | 83 | CTRTDQPCPYDQDCCSGSCTLKKNENGNLVKRC | Shiva Omega superfamily, Omega toxin family (IPR009415;PF06357;) |
| tr|A0A1D0C059|A0A1D0C059\_HADIN | Omega-hexatoxin-Hi1h\_5 | SN\_08\_01 | 45 | 77 | CTPTNQPSPYHESCCSGSCTYKANENGNQVKRC | Shiva Omega superfamily, Omega toxin family (IPR009415;PF06357;) |
| tr|S0F211|S0F211\_HADVN | Omega-hexatoxin-Hvn1b\_1 insecticidal toxin | SN\_08\_01 | 51 | 83 | CIPSGQPCPYNENCCSKSCTYKENENGNTVQRC | Shiva Omega superfamily, Omega toxin family (IPR009415;PF06357;) |
| tr|S0F1N1|S0F1N1\_HADVN | Omega-hexatoxin-Hvn1b\_3 insecticidal toxin | SN\_08\_01 | 51 | 83 | CIPSGQPCPYNENCCSKSCTYKENENGNTVQRC | Shiva Omega superfamily, Omega toxin family (IPR009415;PF06357;) |
| sp|S0F1N6|TO1D\_HADIN | Omega-hexatoxin-Hi1d | SN\_08\_01 | 46 | 78 | CIPTGQPCPYNENCCNQSCTYKANENGNQVKRC | Shiva Omega superfamily, Omega toxin family (IPR009415;PF06357;) |
| sp|P0DMQ3|TO1D\_HADMO | Omega-hexatoxin-Hmo1d | SN\_08\_01 | 51 | 83 | CIPSGQPCPYNEHCCSGSCTYKENENGNTVQRC | Shiva Omega superfamily, Omega toxin family (IPR009415;PF06357;) |
| sp|A5A3H3|TO1D\_ATRRO | Omega-hexatoxin-Ar1d | SN\_08\_01 | 45 | 77 | CIPSGQPCPYNENCCSQSCTFKENENGNTVKRC | Shiva Omega superfamily, Omega toxin family (IPR009415;PF06357;) |
| sp|P81596|TO1C\_HADVE | Omega-hexatoxin-Hv1c | SN\_08\_01 | 4 | 36 | CIPSGQPCPYNENCCSQSCTFKENENGNTVKRC | Shiva Omega superfamily, Omega toxin family (IPR009415;PF06357;) |
| sp|A5A3H4|TO1E\_ATRRO | Omega-hexatoxin-Ar1e | SN\_08\_01 | 45 | 77 | CIPSGQPCPYNENCCSKSCTYKENENGNTVQRC | Shiva Omega superfamily, Omega toxin family (IPR009415;PF06357;) |
| sp|P0C2L5|TO1A\_HADIN | Omega-hexatoxin-Hi1a | SN\_08\_01 | 3 | 35 | CTPTDQPCPYHESCCSGSCTYKANENGNQVKRC | Shiva Omega superfamily, Omega toxin family (IPR009415;PF06357;) |
| sp|P0C2L6|TO1B\_HADIN | Omega-hexatoxin-Hi1b | SN\_08\_01 | 4 | 36 | CIPTGQPCPYNENCCSQSCTYKANENGNQVKRC | Shiva Omega superfamily, Omega toxin family (IPR009415;PF06357;) |
| tr|S0F208|S0F208\_HADVE | Omega/Kappa-hexatoxin-Hv1g\_6 insecticidal toxin | SN\_08\_02 | 40 | 74 | CVPVDQPCSLNTQPCCDDATCTQELNENDNTVYYC | Shiva Omega superfamily, Omega kappa toxin family (IPR009415;PF06357;) |
| tr|S0F1N2|S0F1N2\_ATRRO | Omega/Kappa-hexatoxin-Ar1g\_2 insecticidal toxin | SN\_08\_02 | 40 | 74 | CVPVDQPCSLNTQPCCDDATCTQELNENDNTVYYC | Shiva Omega superfamily, Omega kappa toxin family (IPR009415;PF06357;) |
| sp|S0F209|TOK1H\_HADVE | Omega/Kappa-hexatoxin-Hv1h | SN\_08\_02 | 40 | 74 | CVPVDQPCSLNTQPCCDDATCTQERNENGHTVYYC | Shiva Omega superfamily, Omega kappa toxin family (IPR009415;PF06357;) |
| sp|S0F1M6|TOK1G\_ATRRO | Omega/kappa-hexatoxin-Ar1g | SN\_08\_02 | 40 | 74 | CVPVDQPCSLNTQPCCDDATCTQELNENDNTVYYC | Shiva Omega superfamily, Omega kappa toxin family (IPR009415;PF06357;) |
| sp|S0F207|TOK1G\_HADVE | Omega/kappa-hexatoxin-Hv1g | SN\_08\_02 | 40 | 74 | CVPVDQPCSLNTQPCCDDATCTQELNENDNTVYYC | Shiva Omega superfamily, Omega kappa toxin family (IPR009415;PF06357;) |
| tr|S0F1N5|S0F1N5\_HADVE | Omega/Kappa-hexatoxin-Hv1g\_7 insecticidal toxin | SN\_08\_02 | 40 | 72 | CVPVDQPCSLNTQPCCDDATCTQELNENDNTVY | Shiva Omega superfamily, Omega kappa toxin family (IPR009415;PF06357;) |
| tr|S0F214|S0F214\_HADVE | Omega/Kappa-hexatoxin-Hv1g\_5 insecticidal toxin | SN\_08\_02 | 40 | 74 | CVPVDQPCSLNTQPCCDDATCTQELNENDNTVYYC | Shiva Omega superfamily, Omega kappa toxin family (IPR009415;PF06357;) |
| sp|P83588|TOM1A\_MISBR | Omega-actinopoditoxin-Mb1a | SN\_08\_02 | 4 | 38 | CTPSGQPCQPNTQPCCNNAEEEQTINCNGNTVYRC | Shiva Omega superfamily, Omega kappa toxin family (IPR009415;PF06357;) |
| sp|P81792|TX36\_PHONI | Omega-ctenitoxin-Pn4a | SN\_09\_00 | 37 | 87 | CIPRGEICTDDCECCGCDNQCYCPPGSSLGIFKCSCAHANKYFCNRKKEKC | Spider toxin Tx3-6 family |
| sp|P84756|TOX2\_OXYLI | Omega-oxotoxin-Ol1b | SN\_09\_00 | 4 | 44 | CLPKDSTCGDDCDCCEGLHCHCPLRNMLPAILRCSCQSKDD | Spider toxin Tx3-6 family |
| sp|P84014|TX23\_PHORI | Omega-ctenitoxin-Pr2a | SN\_09\_00 | 2 | 52 | CIPRGEICTDDCECCGCDNECYCPIGSSLGIFKCSCAHANKYFCNRKKEKC | Spider toxin Tx3-6 family |
| sp|P61789|TX5A\_HETVE | Omega-sparatoxin-Hv1a | SN\_10\_01 | 4 | 33 | CGWIMDDCTSDSDCCPNWVCSKTGFVKNIC | Huwentoxin-1 family, AU5A subfamily (IPR011696;PF07740;) |
| sp|P61790|TX5B\_HETVE | Omega-sparatoxin-Hv1b | SN\_10\_02 | 3 | 32 | CGWLFHSCESNADCCENWACATTGRFRYLC | Huwentoxin-1 family, AU5B subfamily (IPR011696;PF07740;) |
| sp|P60590|WGRTX\_GRARO | Omega-theraphotoxin-Gr1a | SN\_10\_07 | 51 | 79 | CVRFWGKCSQTSDCCPHLACKSKWPRNIC | Huwentoxin-1 family, GrTx subfamily (IPR011696;PF07740;) |
| sp|P0DJA9|TX15\_GRARO | Omega-theraphotoxin-Gr2a | SN\_10\_08 | 48 | 76 | CLGFMRKCIPDNDKCCRPNLVCSRTHKWC | Huwentoxin-1 family, Gtx1 15 subfamily (IPR011696;PF07740;) |
| sp|P61104|TXH5\_HAPSC | Omega-theraphotoxin-Hs2a | SN\_10\_17 | 52 | 78 | CRWYLGGCSQDGDCCKHLQCHSNYEWC | Huwentoxin-1 family, Hntx 9 subfamily (IPR011696;PF07740;) |
| sp|D2Y2L8|H9F01\_HAPHA | Omega-theraphotoxin-Hhn1e | SN\_10\_17 | 52 | 78 | CRWYLGGCSQDGDCCKHLQRHSNYEWC | Huwentoxin-1 family, Hntx 9 subfamily (IPR011696;PF07740;) |
| sp|D2Y237|H9B01\_HAPHA | Omega-theraphotoxin-Hhn1a 1 | SN\_10\_17 | 52 | 78 | CRWYLGGCSQDGDCCKHLQCHSNYEWC | Huwentoxin-1 family, Hntx 9 subfamily (IPR011696;PF07740;) |
| sp|D2Y2L7|H9B03\_HAPHA | Omega-theraphotoxin-Hhn1a 3 | SN\_10\_17 | 52 | 78 | CRWYLGGCSQDGDCCKHLQCHSNYEWC | Huwentoxin-1 family, Hntx 9 subfamily (IPR011696;PF07740;) |
| sp|D2Y239|H9B02\_HAPHA | Omega-theraphotoxin-Hhn1a 2 | SN\_10\_17 | 52 | 78 | CRWYLGGCSQDGDCCKHLQCHSNYEWC | Huwentoxin-1 family, Hntx 9 subfamily (IPR011696;PF07740;) |
| sp|D2Y2F0|H9A04\_HAPHA | Omega-theraphotoxin-Hhn1f 4 | SN\_10\_17 | 52 | 78 | CRWYLGGCSQDGDCCKHLQCHSNYEWC | Huwentoxin-1 family, Hntx 9 subfamily (IPR011696;PF07740;) |
| sp|D2Y238|H9C01\_HAPHA | Omega-theraphotoxin-Hhn1b | SN\_10\_17 | 52 | 78 | CRWYLGGCSQDGDCCKHLQCHSNYEWC | Huwentoxin-1 family, Hntx 9 subfamily (IPR011696;PF07740;) |
| sp|D2Y2F1|H9D01\_HAPHA | Omega-theraphotoxin-Hhn1c | SN\_10\_17 | 52 | 78 | CRWYLGGCSQDGDCCKHLQCHSNYEWC | Huwentoxin-1 family, Hntx 9 subfamily (IPR011696;PF07740;) |
| sp|D2Y236|H9A01\_HAPHA | Omega-theraphotoxin-Hhn1f 1 | SN\_10\_17 | 52 | 78 | CRWYLGGCSQDGDCCKHLQCHSNYEWC | Huwentoxin-1 family, Hntx 9 subfamily (IPR011696;PF07740;) |
| sp|D2Y2L5|H9E01\_HAPHA | Omega-theraphotoxin-Hhn1d | SN\_10\_17 | 52 | 78 | CRWYLGECSQDGDCCKHLQCHSNYEWC | Huwentoxin-1 family, Hntx 9 subfamily (IPR011696;PF07740;) |
| sp|D2Y2E9|H9A03\_HAPHA | Omega-theraphotoxin-Hhn1f 3 | SN\_10\_17 | 52 | 78 | CRWYLGGCSQDGDCCKHLQCHSNYEWC | Huwentoxin-1 family, Hntx 9 subfamily (IPR011696;PF07740;) |
| sp|D2Y2E8|H9A02\_HAPHA | Omega-theraphotoxin-Hhn1f 2 | SN\_10\_17 | 52 | 78 | CRWYLGGCSQDGDCCKHLQCHSNYEWC | Huwentoxin-1 family, Hntx 9 subfamily (IPR011696;PF07740;) |
| sp|P56676|TXH1\_HAPSC | Mu/omega-theraphotoxin-Hs1a | SN\_10\_23 | 50 | 77 | CKGVFDACTPGKNECCPNRVCSDKHKWC | Huwentoxin-1 family, HwTx I subfamily (IPR011696;PF07740;) |
| sp|D5J6X1|TXO1A\_PELMU | Omega-theraphotoxin-Cc1a | SN\_10\_41 | 52 | 78 | CKYMFGSCGKSDDCCPKLACKRTFNYC | Huwentoxin-1 family, Jztx 36 subfamily (IPR011696;PF07740;) |
| sp|P83480|TXPR1\_THRPR | Beta/omega-theraphotoxin-Tp1a | SN\_10\_54 | 2 | 28 | CRYWLGGCSAGQTCCKHLVCSRRHGWC | Huwentoxin-1 family, ProTx 1 subfamily (IPR011696;PF07740;) |
| sp|P56854|TX482\_HYSGI | Omega-theraphotoxin-Hg1a | SN\_10\_56 | 7 | 33 | CRYMFGGCSVNDDCCPRLGCHSLFSYC | Huwentoxin-1 family, SNX 482 subfamily (IPR011696;PF07740;) |
| sp|P61509|TX1\_APHSP | Omega-theraphotoxin-Asp1f | SN\_12\_06 | 4 | 36 | CVFSCDIEKEGKPCKPKGEKKCTGGWKCKIKLC | Huwentoxin-2 family, TXP1 subfamily (IPR012625;PF08089;) |
| sp|P0DMD9|TXP3\_BRARH | Omega-theraphotoxin-Ba1c | SN\_12\_06 | 54 | 86 | CVFSCDIEKEGKPCKPKGEKKCTGGWKCKIKLC | Huwentoxin-2 family, TXP1 subfamily (IPR012625;PF08089;) |
| sp|P85504|TXP2\_BRARH | Omega-theraphotoxin-Ba1b | SN\_12\_06 | 4 | 36 | CVFSCDIKKEGKPCKPKGEKKCTGGWRCKIKLC | Huwentoxin-2 family, TXP1 subfamily (IPR012625;PF08089;) |
| sp|P61510|TX2\_APHSP | Omega-theraphotoxin-Asp1g | SN\_12\_06 | 4 | 36 | CVFSCDIEKEGKPCKPKGEKKCSGGWKCKIKLC | Huwentoxin-2 family, TXP1 subfamily (IPR012625;PF08089;) |
| sp|P85497|TXP1\_BRARH | Omega-theraphotoxin-Ba1a | SN\_12\_06 | 4 | 36 | CVFSCDIKKEGKPCKPKGEKKCTGGWRCKIKLC | Huwentoxin-2 family, TXP1 subfamily (IPR012625;PF08089;) |
| sp|P49265|TXP1\_BRASM | Omega-theraphotoxin-Bs1a | SN\_12\_06 | 4 | 36 | CVFSCDIEKEGKPCKPKGEKKCSGGWKCKIKLC | Huwentoxin-2 family, TXP1 subfamily (IPR012625;PF08089;) |
| sp|B3FIV1|TXBS1\_BRASM | Omega-theraphotoxin-Bs2a | SN\_14\_08 | 58 | 93 | CIGESVPCDKDDPRCCREYECLKPTGYGWWYASYYC | Magi-1 superfamily, Ltx4 family (IPR012627;PF08092;) |
| sp|P0CI04|TX132\_APHSP | Omega-theraphotoxin-Asp3a | SN\_14\_08 | 1 | 35 | CLGENVPCDKDRPNCCSKYECLEPTGYGRCYASYY | Magi-1 superfamily, Ltx4 family (IPR012627;PF08092;) |
| tr|A0A1D0BNC2|A0A1D0BNC2\_HADIN | Omega-hexatoxin-Hi2g\_2 | SN\_15\_02 | 22 | 86 | CGKINEDFMENGLESHALHDEIRKPIDTEKADAERGVVGCVLNTLGCSSDKDCCGMTPSCTLGIC | Magi-5 family, Omega atracotoxin type 2 family (IPR012628;IPR013139;PF08093;) |
| sp|Q9BJV8|TOT2A\_ATRIL | Omega-hexatoxin-Asp2a | SN\_15\_02 | 22 | 84 | CGMKNEDFMEKGLESNELHDAIKKPVNSGKPDTERLLDCVLSRVCSPDANCCGLTPICKMGLC | Magi-5 family, Omega atracotoxin type 2 family (IPR012628;IPR013139;PF08093;) |
| tr|A0A1D0C005|A0A1D0C005\_HADIN | Omega-hexatoxin-Hi2g\_1 | SN\_15\_02 | 22 | 86 | CGKINEDFMENGLESHALHDEIRKPIDTEKADAERGVVGCVLNTLGCSSDKDCCGMTPSCTLGIC | Magi-5 family, Omega atracotoxin type 2 family (IPR012628;IPR013139;PF08093;) |
| tr|A0A1D0BR69|A0A1D0BR69\_HADIN | Omega-hexatoxin-Hi2e\_1 (Fragment) | SN\_15\_02 | 21 | 85 | CGKMNEDFMENGLESHALHDEIRKPIDTEKADAERGVLDCVVNTLGCSSDKDCCGMTPSCTLGIC | Magi-5 family, Omega atracotoxin type 2 family (IPR012628;IPR013139;PF08093;) |
| sp|Q9BJV9|TOT2A\_HADIN | Omega-hexatoxin-Hi2a | SN\_15\_02 | 22 | 86 | CGKINEDFMENGLESHALHDEIRKPIDTEKADAERGVLDCVVNTLGCSSDKDCCGMTPSCTLGIC | Magi-5 family, Omega atracotoxin type 2 family (IPR012628;IPR013139;PF08093;) |
| sp|Q9BJV7|TOT2B\_ATRIL | Omega-hexatoxin-Asp2b | SN\_15\_02 | 22 | 84 | CGMKNEDFMEKGLESNELHDAIKKPVNSGKPDTERLLDCVLSRVCSSDANCCGLTPTCKMGLC | Magi-5 family, Omega atracotoxin type 2 family (IPR012628;IPR013139;PF08093;) |
| tr|A0A1D0BPM2|A0A1D0BPM2\_HADIN | Omega-hexatoxin-Hi2f\_1 | SN\_15\_02 | 22 | 86 | CGKINGDFMENGLESHALHDEIRKPIDTEKADAERGLVDCVLNTLGCSSDKDCCGMTPSCTLGIC | Magi-5 family, Omega atracotoxin type 2 family (IPR012628;IPR013139;PF08093;) |
| tr|A0A1D0BN54|A0A1D0BN54\_HADIN | Omega-hexatoxin-Hi2a\_2 | SN\_15\_02 | 22 | 86 | CGKINEDFMENGLESHALHDEIRKPIDTEKADAERGVLDCVVNTLGCSSDKDCCGMTPSCTLGIC | Magi-5 family, Omega atracotoxin type 2 family (IPR012628;IPR013139;PF08093;) |
| sp|Q9BJW0|TOT2B\_HADIN | Omega-hexatoxin-Hi2b | SN\_15\_02 | 22 | 86 | CGKINEDFMENGLESHALHDEIRKPIDTEKADAERGVVDCVLNTLGCSSDKDCCGMTPSCTLGIC | Magi-5 family, Omega atracotoxin type 2 family (IPR012628;IPR013139;PF08093;) |
| sp|P82852|TOT2A\_HADVE | Omega-hexatoxin-Hv2a | SN\_15\_02 | 1 | 29 | LLACLFGNGRCSSNRDCCELTPVCKRGSC | Magi-5 family, Omega atracotoxin type 2 family (IPR012628;IPR013139;PF08093;) |
| sp|P81694|TXC1\_CUPSA | Omega-ctenitoxin-Cs1a | SN\_19\_04 | 49 | 91 | CIPKHEECTNDKHNCCRKGLFKLKCQCSTFDDESGQPTERCAC | Spider toxin CsTx superfamily, U1 lycotoxin family (IPR019553;IPR011142;PF10530;) |
| sp|A9XDG5|TLCOE\_GEOA2 | Omega-lycotoxin-Gsp2671e (Fragment) | SN\_29\_00 | 19 | 59 | CITWRNSCVHNDKGCCFPWSCVCWSQTVSRNSSRKEKKCQC | Omega lycotoxin family |
| sp|A9XDG0|TLCOB\_GEOA2 | Omega-lycotoxin-Gsp2671b | SN\_29\_00 | 44 | 84 | CITWRNSCMHNDKGCCFPWSCVCWSQTVSRNSSRKEKKCQC | Omega lycotoxin family |
| sp|A9XDG3|TLCOG\_GEOA2 | Omega-lycotoxin-Gsp2671g | SN\_29\_00 | 44 | 84 | CITWRNSCMHYDKGCCFPWTCVCWSQTVSRNSSRKEKKCQC | Omega lycotoxin family |
| sp|A9XDG2|TLCOD\_GEOA2 | Omega-lycotoxin-Gsp2671d | SN\_29\_00 | 44 | 84 | CITWRNSCMHNDKGCCFPWSCVCWSQTVPRNSSRKEKKCQC | Omega lycotoxin family |
| sp|A9XDG4|TLCOF\_GEOA2 | Omega-lycotoxin-Gsp2671f (Fragment) | SN\_29\_00 | 22 | 62 | CTTWRNSCMHNDKGCCFPWSCVCWSQTVSRNSSRKEKKCQC | Omega lycotoxin family |
| sp|P85079|TLCOA\_GEOA2 | Omega-lycotoxin-Gsp2671a | SN\_29\_00 | 44 | 84 | CITWRNSCMHNDKGCCFPWSCVCWSQTVSRNSSRKEKKCQC | Omega lycotoxin family |
| sp|A9XDG1|TLCOC\_GEOA2 | Omega-lycotoxin-Gsp2671c | SN\_29\_00 | 44 | 84 | CITWRNSCMHNDKGCCFPWSCVCWSQTVSRNSSGKEKKCQC | Omega lycotoxin family |
| sp|P83476|TXPR2\_THRPR | Beta/omega-theraphotoxin-Tp2a | SN\_30\_00 | 2 | 25 | CQKWMWTCDSERKCCEGMVCRLWC | Phrixotoxin family |
| sp|P68424|TXH10\_HAPSC | Omega-theraphotoxin-Hs1a | SN\_36\_00 | 13 | 67 | CLVVSTHAERHSKTDMEDMEDSPMIQERKCLPPGKPCYGATQKIPCCGVCSHNKC | Huwentoxin-Type-10 |

  
  
